# Value of clinical examination in the assessment of penetrating neck injuries: a retrospective study of diagnostic accuracy test

**DOI:** 10.1186/s12873-020-00311-4

**Published:** 2020-03-09

**Authors:** Andrés Isaza-Restrepo, Julián Andrés Quintero-Contreras, Jorge Escobar-DiazGranados, Ángela María Ruiz-Sternberg

**Affiliations:** 1 Escuela de Medicina y Ciencias de la Salud. Grupo de Investigación Clínica, Universidad del Rosario, Carrera 24 No 63C-69 Barrio Siete de Agosto, Bogotá, DC Colombia; 2 Méderi Hospital Universitario Mayor, Bogotá, DC Colombia; 3 Hospital Emiro Quintero Cañizares, Ocaña, Colombia

**Keywords:** Penetrating neck injuries, Vascular, Airway and gastrointestinal signs, Health resources

## Abstract

**Background:**

There are many high-volume trauma centers in limited resource environments where a thorough clinical examination of patients may contribute to a more economical, accurate, and widely applicable method of determining the proper management of patients with penetrating neck injuries. The purpose of this study was to validate thorough physical examination as a reliable diagnostic tool in these patients.

**Methods:**

We performed an observational retrospective study of a diagnostic accuracy test where we compared clinical findings (symptoms and soft signs on admission of the patient) with the definitive findings according to the gold standard test for each particular situation (selective studies, clinical observation and surgical exploration). The study was conducted at Hospital Occidente Kennedy (HOK) between August 2009 and June 2010.

**Results:**

The sample consisted of the clinical records of 207 (*n* = 207) patients who went to the emergency room for penetrating neck wounds at Hospital Occidente Kennedy (HOK). Of the total sample, 36.2% (*n* = 75) of patients were considered “asymptomatic” as they didn’t present with any soft signs of injury. Vascular soft signs were present in 57% (*n* = 118) of the patients, soft signs of the airway and the upper gastrointestinal tract were present in 15.9% (*n* = 33) and 21.3% (*n* = 44) of the patients respectively. The sensitivity and negative predictive value (NPV) of any soft sign to determine injuries which require surgical repair was 97.4% [CI] [86.5–99.5%] and 98.7% [CI] [92.8–99.8%] respectively, with a range of confidence [CI] of 95%.

**Conclusions:**

Our study’s main findings suggest that patients with neck injuries and no vascular, airway, or gastrointestinal soft sign can be safely managed with a conservative approach. It is important to emphasize the value of the clinical examination since there are many contexts in the modern world where a considerable amount of the population is afflicted by neck trauma and treated under conditions where technological resources are limited.

## Background

The neck has a dense concentration of vital structures confined in a small space. Any intrusion may result in life-threatening penetration of the trachea or larynx, the esophagus, major blood vessels, cervical nerve roots, cervical nerves or the spinal cord [1, 2]. Penetrating neck injury (PNI) constitutes 5–10% of traumatic injuries in adults and has a mortality rate ranging from 10 to 15% [1, 2]. Different characteristics of neck trauma are usually related to the social and cultural conditions of the population. Penetrating neck injuries are more likely to occur in highly populated areas where there is more interpersonal violence and more gun and knife crime between young men [3]. Additionally, the rise of global terrorism and continuing wars in the Middle East has perpetuated head and neck injuries in the civilian population leading to elevated morbidity and mortality rates [4, 5].

Previously many centers advocated for mandatory surgical exploration irrespective of signs or symptoms. Such policies are associated with a high incidence of unnecessary operations, ranging from 30 to 89% [6]. Different studies suggest operative management is required more frequently with gunshot wounds than with stab wounds, even though the majority of PNIs are caused by stab wounds, with only a minority by gunshot injuries [3, 7]. In recent years, selective management of penetrating neck trauma based on clinical examination has been recommended. This has seemed to reduce, on the one hand, the percentage of negative examinations, and on the other hand, a significant number of costly diagnostic tests, many of which are not available in some institutions, which also generate unnecessary prolongation of hospital stays [6].

However, management of PNI remains controversial. Over the last decade, most trauma centers have now adopted the approach of selective management based on a combination of clinical examination and the use of diagnostic tools [8]. In particular, developments in computed tomography angiography (CTA) have provided a fast, accurate, non-invasive method for evaluating patients with penetrating neck injuries [8].

Although, in settings where resources are limited and this technology is not yet available, other types of studies (angiography, bronchoscopy and/or esophagoscopy) still hold high sensitivity for detecting injuries. These exams are also invasive for the patient and carry a small but serious risk of complications [9]. The high incidence of PNIs due to interpersonal violence within cities and due to the continuation of wars in the Middle East raises questions as to whether patients should be managed by using CTA as the gold standard diagnostic approach or if efforts must be focused on the rational use of technology. This being even more so when the value of the clinical examination plays a very important role defining the management of the patient [8, 9].

The current trend in literature is to argue the selective use of different diagnostic methods depending on the clinical findings upon admission [10]. High-volume trauma centers are more frequently turning to the selective non-operative management (SNOM) approach when treating PNIs. This type of management is based on clinical examination or other additional examinations. Together, they have shown to be reliable indicators of clinically significant injury, with a sensitivity of 93–95% and a negative predictive value of 97% [10, 11].

Bearing in mind the changes in the management of PNIs, our study aims to provide information on the value of the clinical examination in the assessment of penetrating neck wounds, in scenarios where there is a high volume of trauma and there are limited resources for decision making. To this end, a diagnostic accuracy test study was designed to evaluate the diagnostic value of clinical findings in neck trauma (to identify the presence of lesions that warranted further study or surgical intervention). Our research shows the experiences within a trauma referral center with limited resources in Bogotá, Colombia. In this study, we aimed to evaluate the diagnostic accuracy of clinical findings in patients with penetrating neck injuries compared with definitive findings found trough gold standard test, clinical follow-up and/or surgical exploration and to determine its diagnostic potential in patients attended in limited resource settings.

## Methods

We performed an observational retrospective study to compare the diagnostic accuracy of clinical findings (symptoms and soft signs on admission of the patient) with the definitive findings according to the gold standard test for each particular situation in patients with PNI admitted to the Hospital Occidente Kennedy’s (HOK) emergency department from August 2009 until and including June 2010. Kennedy is a middle to low income borough in Bogota. It is the second largest borough in the city, with approximately one million inhabitants, from which its majority belongs to an age group between 25 to 29 years old [12]. Between 2009 and 2010 this locality accounted for 12% of the felonies in the city and had the highest number of cases of theft, traffic accidents and homicides [13]. When information was gathered, approximately 2000 trauma patients were admitted in HOK facilities annually, with 8% of the cases being associated with penetrating neck injuries. We determined the diagnostic accuracy of clinical findings following the Standards for Reporting Diagnostic accuracy studies (STARD) 2015 guidelines [14].

Patients admitted to the HOK emergency department with neck wounds that extended deep to the platysma were considered for inclusion. The selected group was between 14 and 65 years of age. They underwent an interview and a complete physical examination, and a form was filled out by the surgeon on duty, where he/she focused on soft signs and symptoms that would lead the physician to suspect lesions in the upper gastrointestinal tract, airway lesions in the neck and/or vascular lesions. Patients with penetrating neck trauma who were initially resuscitated according to the Advanced Trauma Life Support guidelines and remained hemodynamically unstable were excluded from the study [15]. Patients were also excluded and taken immediately to the operating room if they presented “hard signs” including signs of vascular injury (active severe bleeding, expanding hematoma, absence of peripheral pulse, arterial bruit, thrill, and unexplained hypotension) and aerodigestive injury (respiratory distress, massive subcutaneous emphysema, air bubbling through the neck wound and massive hemoptysis) [15]. Additionally, patients were excluded if they were dead on arrival (DOA), those with insufficient data in the clinical record or without clinical follow-up, and patients with cranioencephalic and/or spinal cord trauma of whom, due to their clinical condition, necessary information couldn’t be collected.

After a comprehensive clinical evaluation, patients included in the research were categorized into two groups according to their signs and symptoms. The first group consisted of patients with “soft signs” including signs of vascular injury (minimal bleeding, mild to moderate bruising, hypotension that responds to resuscitation, and murmur fluids) and aerodigestive injury (hoarseness, stridor, minimal hemoptysis, subcutaneous emphysema, odynophagia, dysphagia and hematemesis) [15]. The second group was comprised of patients who did not present any hard or soft signs upon clinical examination, and so were considered “asymptomatic” (however still presenting with PNI) and would undergo close observation and series of physical tests for 24 h.

To determine whether patients required any surgical repair, we used three gold standard tests. The surgeon on duty decided between three different types of patient management according to the initial clinical findings: 1. Request selective studies: Fibrobronchoscopy (airway), arteriography and/or duplex studies (vascular), upper digestive endoscopy (upper gastrointestinal tract). 2. Surgical exploration: intraoperative findings. 3. Clinical observation: patients that the surgeon on duty left in clinical observation for 24 h in the emergency service and hereafter were discharged. Subsequently, a follow-up was carried out by external consultation or a telephone call 30 days after the trauma where a questionnaire was carried out to determine if the patient presented late signs or symptoms that required complementary studies, or if they were asymptomatic, at which point the patients were considered to be fully recovered.

Clinical findings were compared using two-by-two contingency tables, the accuracy of the soft signs of vascular, airway and digestive injury was estimated comparing patients who required surgery with patients who didn’t (according to the gold standard in each patient). Additionally, 95% confidence intervals (CI) were calculated. Data was collected in a pre-established format and analyzed with the statistical packages STATA version 9 and SPSS version 17. A descriptive analysis of the variables was carried out, and sensitivity, specificity, positive and negative predictive values of clinical examination were calculated.

## Results

The sample consisted of the clinical records of 207 (*n* = 207) patients who were admitted for penetrating neck wounds at HOK between August 2009 and June 2010 and met the inclusion criteria. The majority were male, the mean age was 29.22 years (SD:11.92). The most frequent neck injuries were caused by stab wounds and mainly affected the zone II of the neck (Table 1).

**Table 1:**
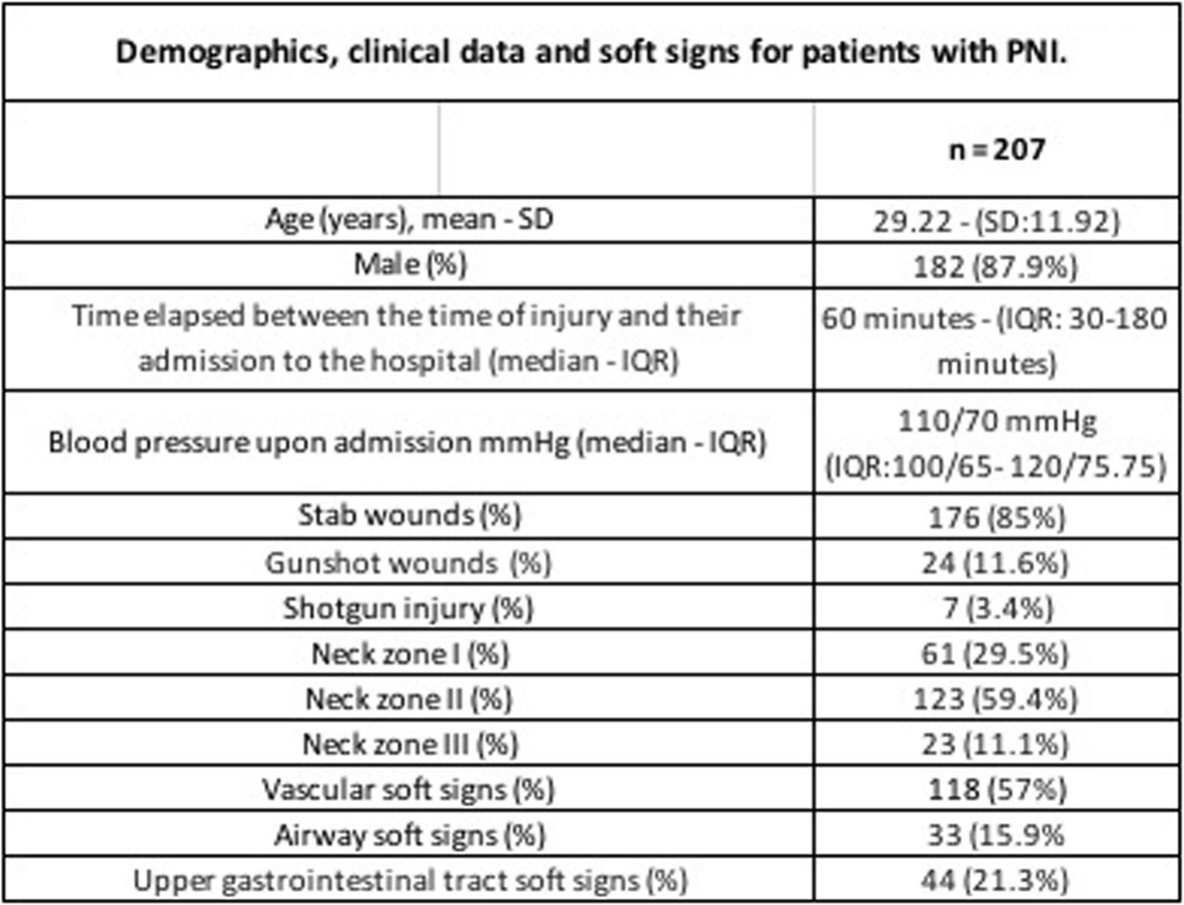
Demographics, clinical data and soft signs for patients with PNI.

**Fig. 1:**
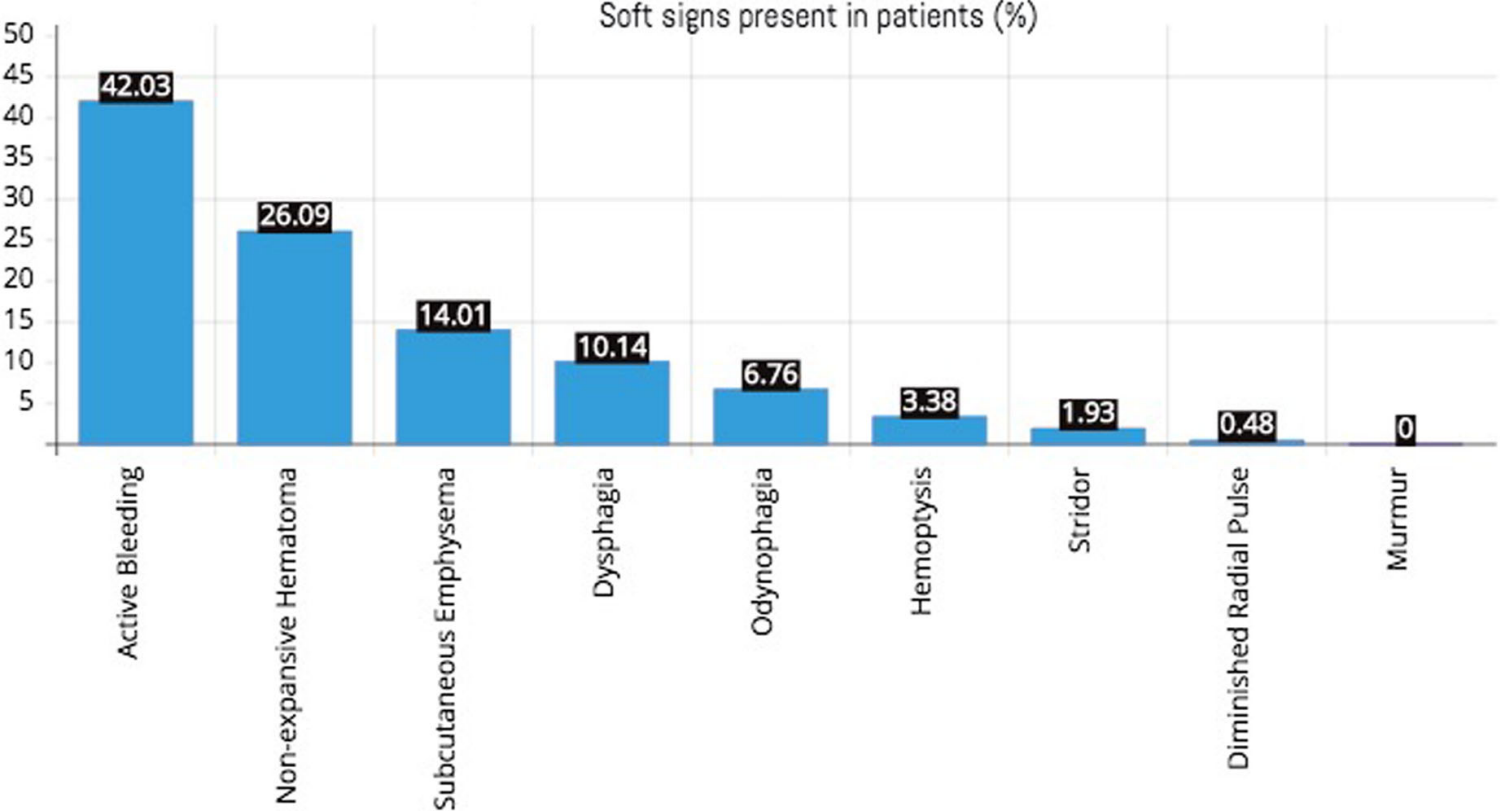
Soft signs present in patients (%)

Vascular soft signs were present in 118 (57%) patients, 87 (42%) patients presented minimal bleeding, followed by 54 (26%) who presented non-expansive hematomas. The soft signs of the airway and the upper gastrointestinal tract were present in 33 (15.9%) and 44 (21.3%) patients, respectively. With regards to the airway and in the upper gastrointestinal tract, the most frequent sign was subcutaneous emphysema present in 29 (14.1%) patients. Hemoptysis occurred in only 7 patients and stridor in 4 patients. Dysphagia occurred in 21 (10.1%) patients and odynophagia in 14 (6.8%) (Fig. 1).

Regarding the diagnostic and/or therapeutic approach of the 207 patients, 95 (45.9%) underwent the set of selective studies. Upper digestive tract endoscopy was performed in 95 patients and did not show evidence of any lesion. Only one patient had an abnormal fibrobronchoscopy, underwent surgical exploration and required surgical repair of the trachea (although this patient presented with a vascular soft sign). Carotid duplex was performed in 80 patients and only one patient needed surgical repair (internal carotid stent). Additionally, of the 22 arteriographic studies performed, only one patient showed evidence of lesions that required surgical repair (internal carotid stent), while another patient had a vertebral artery thrombosis which was managed during the procedure.

Furthermore, from the 83 (40.1%) patients who underwent surgical exploration, 60 (72.3%) patients showed no lesions of vital structures. Some type of vascular lesion was identified in 23 (27.7%) patients (total or partial section of the anterior or external jugular veins; in three cases evidence was found of injuries to the internal carotid and the internal jugular). Airway injury was identified in 16 (20%) patients; all cases were characterized by perforations of the trachea. No injury of the upper gastrointestinal tract was identified in this series of patients. From the 75 (36.2%) patients categorized in the “asymptomatic” group (as they didn’t present any soft sign of injury), 37 (49.3%) had complementary studies where only one needed surgical repair (internal carotid stent). Additionally, 11 (14.6%) patients were taken to surgery and none required any repair. Of the 207 patients, only 30 (14.5%) patients had clinical follow-up, and none required further assessment.

**Fig. 2:**
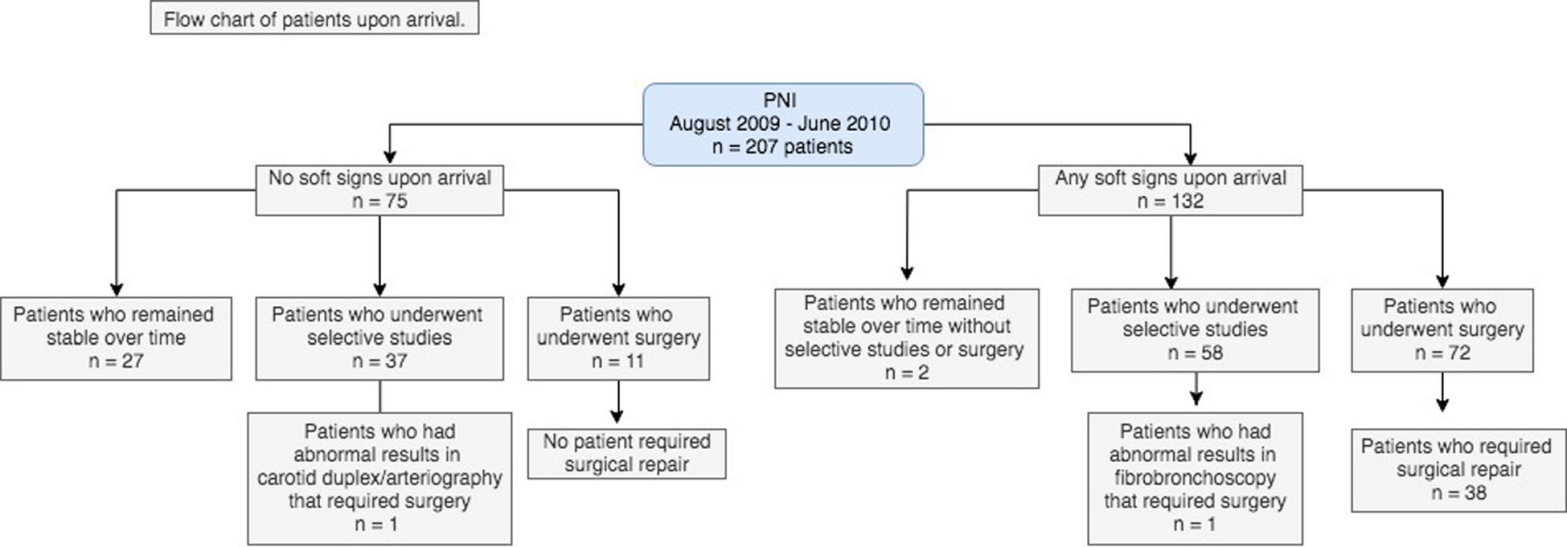
Flow chart of patients upon arrival

Of the 207 patients, only 38 (18.4%) had injuries that required surgical repair: 23 (11.1%) of the patients presented vascular lesions and 16 (7.7%) airway lesions. Only one patient needed repair of a vascular and airway injury (external jugular and trachea). A flow chart is shown to represent the management of the 207 patients upon arrival (Fig. 2).

### Analytical statistics

The validation analysis showed in Table 2 demonstrates that the clinical finding of any soft sign has a sensitivity of 97.37%, with a specificity 43.79%, a positive predictive value (PPV) of 28.03% and a negative predictive value (NPV) of 98.67% to determine which patients required surgical repair. Two-by-two contingency Tables 3, 4 and 5 represent clinical findings on vascular, airway and any soft sings from which the results in Table 2 come.

**Table 2:**
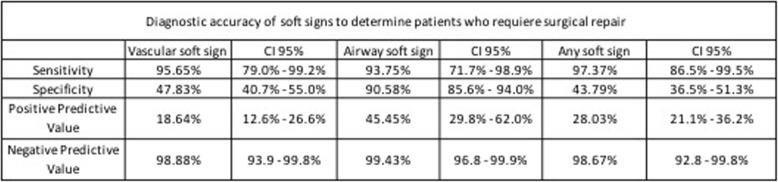
Diagnostic accuracy of soft signs to determine patients who requiere surgical repair.

**Table 3:**
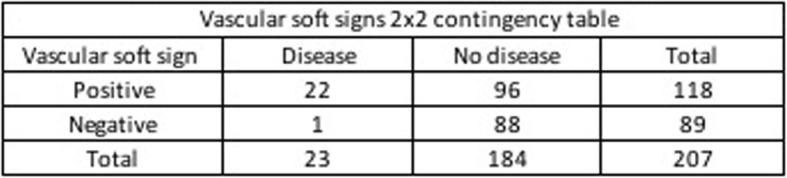
Vascular soft signs 2 × 2 contingency table.

**Table 4:**
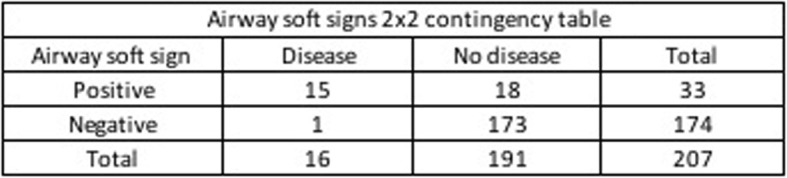
Airway soft signs 2 × 2 contingency table.

**Table 5:**
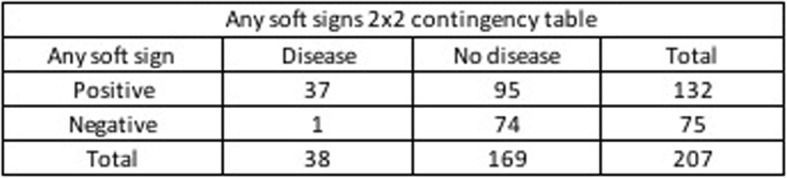
Any soft signs 2 × 2 contingency table.

Clinical findings of vascular and airway soft signs demonstrated high diagnostic value to identify injuries that require surgical repair. Diagnostic accuracy was calculated with a range of confidence of 95% [CI].

## Discussion

Our study’s main findings suggest that in settings with limited resources, patients with PNI and no vascular, airway or gastrointestinal soft sign can be safely managed with a conservative approach. These hold high negative predictive values in the absence of soft sings (NPV: 93–99%). Our findings also demonstrate that adequate identification of soft signs represents a reasonable screening tool for patients with PNIs, as it shows high sensitivity (97.4%) [CI] [86.5–99.5%] in determining patients who require surgical repair. Many high-volume trauma centers are using the Selective Non-operative Management Approach (SNOM) [7]. These works by determining the soft signs of vascular injury, airway injury and upper gastrointestinal tract lesion. SNOM help suspect vital structure lesions and to identify the need to carry out complementary studies [7]. Similar to our results, the study of Inaba et al. with 453 patients at two Level 1 trauma centers demonstrate that using physical examination to triage patients into those with hard signs, soft signs, and no signs of vascular and aerodigestive tract injuries was highly effective at minimizing the need for invasive imaging and the rate of negative surgical exploration [16]. Different studies validated the practice of SNOM as a safe an appropriate management strategy [7, 17].

Identification of vascular soft signs evidenced high sensitivity (95.6%) [CI] [79–99.2%] and NPV (98.9%) [CI] [93.9–99.8%] in order to determine patients who require surgical repair. The possibility of requiring surgical repair if one does not present any vascular soft sign is low. While some authors advocate mandatory vascular imaging following PNIs according to mechanism or zone, many studies have demonstrated the reliability of physical examination alone to exclude clinically relevant vascular injuries [11, 18]. The no zone approach has become more relevant as identification of soft signs hold higher value to identify lesions that require repair, regardless of the zone of the neck injured [17]. A prospective observational study with 203 patients showed a negative predictive value (NPV) and specificity of 88.6–95.5% and 90.7% respectively, for all signs and symptoms mandating angiography [18]. Additionally, in a study conducted with 216 patients in Indiana the sensitivity and negative predictive value of physical examination for detecting vascular injuries requiring operative management were both 100% [11]. Moreover, the research of Menawat et al. conducted with 110 patients evidenced that 42 patients were determined to have no vascular injury based on lack of any physical findings [19]. According to our findings and evidence shown in literature it is safe to say that routine angiography may be unnecessary for patients with penetrating neck injuries and negative physical examination.

Taking into account soft signs of airway injury, our study showed a sensitivity of 93.8% [CI] [71.7–98.9%], a specificity of 90.6% [CI] [85.6–94%], and a NPV of 99.4% [CI] [96.8–99-9%] to diagnose airway injuries that required surgical repair. Patients presenting airway soft signs may be evaluated with bronchoscopy, esophagogram and esophagoscopy [11]. Nevertheless, new technologies such as CTA have demonstrated to hold high diagnostic accuracy when compared to the vascular and airway diagnostic gold standards. Different studies showed nearly 100% sensitivity and specificity of CTA in detecting clinically significant vascular or aerodigestive injuries [17, 20–22].

As shown, any vascular or airway soft sign identified in patients should impact the management received. Different diagnostic studies (Arteriography, duplex of four vessels of the neck, bronchoscopy, esophagogram, esophagoscopy and more recently CTA) have shown high sensitivity and negative predictive values to determine injuries in patients with PNI [11, 21]. Although, we should bear in mind the significant resource implications associated with unnecessary utilization of diagnostic tools [16]. In a resource-limited environment, unnecessary procedures may be interpreted as the loss of opportunity in the care of many patients, which is why the confirmation of the safety and accuracy of physical examination in patients with PNI represents a valuable strategy towards a simpler, more economic, accurate, and widely applicable method of determining the proper management of these patients [23].

A consensus regarding the management of penetrating neck injuries has been sought around the world. It is clear that immediate neck exploration is warranted in unstable patients with “hard signs” of neck injury after optimal airway has been obtained [15]. In accordance to international literature, our study showed higher incidence of stab wounds than gunshot wounds as a result of interpersonal violence within city limits [3, 7, 17, 19, 23]. Nevertheless, higher incidence of gunshot wounds is evidenced in scenarios of war and armed conflict. Additionally, according to literature there’s a higher prevalence of PNIs in men, and the most affected zone of the neck is usually zone II [17–19, 23]. However, the type of management of the PNI shouldn’t be determined by the mechanisms of assault or neck zone affected but by the signs and symptoms presented by the patient [3, 4, 11, 18].

There are some limitations to this study that need to be emphasized. Primarily, this was an observational retrospective study. If we wish for there to be a future paradigm shift towards a SNOM approach in scenarios of low resources, a prospective study would be more appropriate. Also, a bigger sample size could help corroborate our results. Additionally, the hospital where the study was conducted did not have available CTA imaging. As different studies showed a nearly 100% accuracy in detecting vascular and aerodigestive injuries, it would have been important to count on this diagnostic tool as a gold standard.

## Conclusions

It is important to emphasize the value of clinical examination considering the many contexts in which the population is subjected to neck trauma and technological resources are limited. The loss of resources in unnecessary procedures, may be interpreted as the loss of opportunity of caring for many other patients in a resource-limited environment. The results of the present study indicates that physical examination is a reliable, simpler, economical, accurate, and widely applicable diagnostic tool. Furthermore, it can be specifically implemented and used systematically to diagnose the patient’s signs and/or symptoms related to lesions of the digestive tract, vascular system, airway, and nervous system in contexts where more sophisticated technologies are not yet available.

## Data Availability

The datasets used and/or analyzed during the current study are available from the corresponding author on reasonable request.

## Abbreviations

CTA: Computed Tomography Angiography
HOK: Hospital Occidente Kennedy
IQR: Interquartile Range; CI: Confidence Interval
NPV: Negative Predictive Value
PNI: Penetrating Neck Injury
PPV: Positive Predictive Value
SD: Standard Deviation
SNOM: Selective Non-Operative Management
